# *Candidatus* Neoehrlichia mikurensis in Ticks from Migrating Birds in Sweden

**DOI:** 10.1371/journal.pone.0133250

**Published:** 2015-07-24

**Authors:** Lisa Labbé Sandelin, Conny Tolf, Sara Larsson, Peter Wilhelmsson, Erik Salaneck, Thomas G. T. Jaenson, Per-Eric Lindgren, Björn Olsen, Jonas Waldenström

**Affiliations:** 1 Uppsala University, Uppsala, Sweden; 2 Department of Infectious Diseases, Kalmar County Hospital, Kalmar, Sweden; 3 Center for Ecology and Evolution in Microbial Model Systems (EEMiS), Linnaeus University, Kalmar, Sweden; 4 Linköping University, Linköping, Sweden; University of Minnesota, UNITED STATES

## Abstract

*Candidatus* Neoehrlichia mikurensis (CNM; family *Anaplasmataceae*) was recently recognized as a potential tick-borne human pathogen. The presence of CNM in mammals, in host-seeking *Ixodes* ticks and in ticks attached to mammals and birds has been reported recently. We investigated the presence of CNM in ornithophagous ticks from migrating birds. A total of 1,150 ticks (582 nymphs, 548 larvae, 18 undetermined ticks and two adult females) collected from 5,365 birds captured in south-eastern Sweden was screened for CNM by molecular methods. The birds represented 65 different species, of which 35 species were infested with one or more ticks. Based on a combination of morphological and molecular species identification, the majority of the ticks were identified as *Ixodes ricinus*. Samples were initially screened by real-time PCR targeting the CNM *16S* rRNA gene, and confirmed by a second real-time PCR targeting the *groEL* gene. For positive samples, a 1260 base pair fragment of the *16S* rRNA gene was sequenced. Based upon bacterial gene sequence identification, 2.1% (24/1150) of the analysed samples were CNM-positive. Twenty-two out of 24 CNM-positive ticks were molecularly identified as *I*. *ricinus* nymphs, and the remaining two were identified as *I*. *ricinus* based on morphology. The overall CNM prevalence in *I*. *ricinus* nymphs was 4.2%. None of the 548 tested larvae was positive. CNM-positive ticks were collected from 10 different bird species. The highest CNM-prevalences were recorded in nymphs collected from common redpoll (*Carduelis flammea*, 3/7), thrush nightingale (*Luscinia luscinia*, 2/29) and dunnock (*Prunella modularis*, 1/17). The *16S* rRNA sequences obtained in this study were all identical to each other and to three previously reported European strains, two of which were obtained from humans. It is concluded that ornithophagous ticks may be infected with CNM and that birds most likely can disperse CNM-infected ticks over large geographical areas.

## Introduction

*Candidatus* Neoehrlichia mikurensis (CNM) is a recently recognized tick-transmitted potential human pathogenic rickettsia of the family *Anaplasmataceae*. This obligately intracellular gram-negative bacterium is transmitted to humans by ticks. It was first described after being found in *Ixodes ovatus* and isolated from brown rats (*Rattus norvegicus*) in Japan in 2004 [1]. However, it was later realized that this bacterium had previously been detected in *I*. *ricinus* in the Netherlands but was at that time referred to as an *Ehrlichia*-like species [2]. In 2010, the first human CNM-infected patient was described in Sweden [3]. So far, 14 human cases have been reported in Europe, predominantly in immunocompromised patients [4–6]. Another seven human cases have been reported in China, all in previously healthy individuals [7]. The symptoms include persistent fever, chills, arthralgia, myalgia, headache and thromboembolic events. One patient died from the CNM infection [8]. Diagnosis is currently based on molecular detection of the CNM *16S* rRNA gene in blood [9], since no isolation technique has been established.

In the last years, studies conducted in different European countries have shown that the prevalence of CNM in field-collected host-seeking *I*. *ricinus* ticks may range from 0% to 12% [10]. The CNM prevalence in The Netherlands was reported as 6, 8, 4 and 13% in adult *I*. *ricinus* removed from red deer (*Cervus elaphus*), wild boar (*Sus scrofa*), sheep (*Ovis aries*) and mouflon (*Ovis orientalis musimon)*, respectively [8]. CNM was found in 5 of 276 (1.8%) spleens from bank voles (*Myodes glareolus*) in France [11]. In southern Sweden, the prevalence of CNM was 9.0% in *M*. *glareolu*s and 5.7% in *Apodemus* spp. [12]. These studies show that CNM is present in both *I*. *ricinus* and in mammals in Europe. Given the fact that *I*. *ricinus* is commonly retrieved from wild birds, it is important to study the potential role of birds in the epidemiology of CNM. Hypothetically, birds may be competent transmission hosts for CNM or incompetent transmission hosts but yet hosts of ticks, which could be CNM-infected. In both ways it could be conceivable that birds can spread the bacterium to new regions along their migratory flyways [13, 14]. The first record of CNM in a tick infesting a bird is from 2006, when Spitalska *et al*. detected an *Ehrlichia*-like species, referred to as the “Schotti variant”, in an *I*. *ricinus* nymph collected from a song thrush (*Turdus philomelos*) in Slovakia [15]. In 2012, Movila *et al*. screened ticks from migratory birds in the Baltic Region of Russia; CNM was found in 1 of 135 (0.7%) *I*. *ricinus* and in 1 of 4 *I*. *frontalis* [16]. In a recent study from Switzerland, CNM was found in 7 of 215 ticks (3.3%) collected from breeding and migratory birds. All seven CNM-positive ticks were *I*. *ricinus* nymphs. Six of them were collected from four migrant chaffinches (*Fringilla coelebs*) and one nymph was collected from a wren (*Troglodytes troglodytes*) [14].

These studies are limited, but suggest that CNM can be found in ticks feeding on birds. In the present study, a large representative tick material retrieved from birds caught during stopover on spring and autumn migration in South Sweden was screened for CNM. Combining molecular screening for CNM and molecular species identification of ticks, we aimed to provide a better baseline for the presence of CNM in birds. If birds act as transmission hosts of CNM to blood-feeding ticks, we hypothesize that CNM should be detected in all active stages of strictly ornitophagous (bird-feeding) ticks and in tick larvae of species with a broader host range retrieved from birds.

## Materials and Methods

### Bird trapping

All birds were trapped during the normal trapping activities of staff members at the Ottenby Bird Observatory (56° 12′ N, 16° 24′ E), Sweden, under a general ringing license from the Swedish Ringing Office (Ringmärkningscentralen, Naturhistoriska riksmuseet) in accordance with Swedish regulations. Sampling of birds was approved by the Swedish Board of Agriculture, delegated through Linköpings Djurförsöksetiska nämnd in Linköping (decision 43–09).

### Tick collection

During 2009, 5,365 migratory birds caught at Ottenby Bird Observatory were examined for ticks around their bills and eyes and in the ears. The site of attachment on the bird was noted for each tick before it was removed, photographed for identification of species and stage, and stored at -70°C until further investigation. In total, 1,335 ticks from 748 birds were collected. Of these, 21 ticks could not be removed from the birds and 162 ticks were lost due to technical problems during nucleic acid extraction, resulting in 1,150 ticks available for analysis.

### Tick identification

Each tick was photographed dorsally and ventrally using a digital USB-microscope, Dino-Lite Long AM4013TL (AnMo Electronics Corp., Taiwan). Based on photographs and the descriptions in [17–20], the ticks were morphologically identified, *i*.*e*. the developmental stage (larva, nymph or adult), sex of adults and genus of larvae, nymphs and adults were recorded. The degree of blood ingested by each tick was estimated as unfed (U), little fed (LF; a relatively small amount of host blood in the tick's gut), half fed (HF) or fully fed (FF). The digital photographs did not always capture the distinguishing criteria, or the tick had been damaged during removal from the birds. Determination of the species was therefore often uncertain or even impossible. To remedy this, all nymphs and adults were further screened by molecular methods. The cDNA from the CNM screening was used as template in the PCR reactions. The first method targeted the mitochondrial gene cytochrome C oxidase subunit I (*COI*), which was amplified by the primers Cox1F and Cox1R (Table 1) [21]. The PCR reactions were carried out in 25 μl reaction volumes, containing 2 μl of template cDNA, 10 μM of forward and reverse primers, 5 μl Q-solution, 2.5 μl 1x PCR buffer (Applied Biosystems, The Netherlands), 0.2mM deoxynucleoside triphosphates, 1.5mM MgCl_2_ and 1.25U of Amplitaq polymerase (Applied Biosystems, The Netherlands). Thermal conditions included incubation at 94°C for 3 min followed by 45 cycles at 95°C for 30 s, 45°C for 30 s and 72°C for 3 min, and a final 7 min elongation at 72°C. Samples that could not be identified with the *COI* method were further analysed with a method that targeted the mitochondrial *16S* rDNA gene. The gene was amplified using the forward primer m16sFW/tck and the reverse primer m16sRev/tck (Table 1) [20]. The PCR reactions were carried out in 25 μl reaction volumes, containing 5ul of template cDNA, 10 μM of forward and reverse primers, 5 μl Q-solution, 2.5 μl 1x PCR buffer (Applied Biosystems, The Netherlands), 0.2mM deoxynucleoside triphosphates, 1.5mM MgCl_2_ and 1.25U of Amplitaq polymerase (Applied Biosystems, The Netherlands). PCR was performed with an initial denaturation step at 94°C for 4 min followed by 35 cycles of 30 s at 92°C, 30 s at 50°C and 45 s at 72°C and a final extension step for 8 min at 72°C. Thermal conditions included incubation at 94°C for 4 min, followed by 35 cycles at 92°C for 30 s, 50°C for 30 s and 72°C for 45 s, and a final 8 min elongation at 72°C. Positive samples were sequenced with the forward primer at a commercial centre (Macrogen Europe, The Netherlands).

**Table 1:** Primers used for screening and sequencing.

Primer name	Target gene	Primer sequence	Reference
Neo_16S_F	*16S* rRNA	5'-GTAAAGGGCATGTAGGCGGTTTAA-3'	Primers were established as part of this study
Neo_16S_R	*16S* rRNA	5'-TCCACTATCCTCTCTCGATCTCTAGTTTAA-3'	Primers were established as part of this study
Neo_16S_95_F	*16S* rRNA	5'-TTAGTGGCAGACGGGTGAGTAATG-3'	Primers were established as part of this study
Neo_16S_127_F	*16S* rRNA	5'-TCTGCCTAGTAGTATGGAATAGCTG-3'	Primers were established as part of this study
Neo_16S_1363_R	*16S* rRNA	5'-AAACCAATTTCCAGGGCATGACGG-3'	Primers were established as part of this study
Neo_16S_1393_R	*16S* rRNA	5'-TCCTTACGGTTAGCTCACCAGCTT-3'	Primers were established as part of this study
NeogroELQf	*GroEL*	5'-ACAGCCAATACTACCTATCCTTGA-3'	Primers, although with a slightly modified version of the reverse primer, were initially reported by Andersson and colleagues [22]
NeogroELQr	*GroEL*	5'-ACATGTAATCCACCACGCAACT-3'	Primers, although with a slightly modified version of the reverse primer, were initially reported by Andersson and colleagues [22]
Cox1F	COI	5´-GGAACAATATATTTAATTTTTGG-3´	[21]
Cox1R	*COI*	5´-ATCTATCCCTACTGTAAATATATG-3´	[21]
m16sFW/tck	*16S* rRNA	5´-CCGGTCTGAACTCAGATCAAGT-3´)	[20]
m16sRev/tck	*16S* rRNA	5´-GCTCAATGATTTTTTAAATTGCTGT-3´)	[20]

### Molecular CNM detection, sequencing and phylogenetic analysis

Extracting total nucleic acid (DNA and RNA) from a tick allows for the monitoring of known and emerging vector-borne pathogens. Therefore, after total nucleic acid extraction and cDNA synthesis as previously described [23], cDNA was screened using a real-time PCR protocol including specific primers targeting the CNM *16S* rRNA-gene (Table 1) to generate 107 bp DNA fragments using the 2X iQ SYBR Green supermix (Bio-Rad Laboratories, USA) on either a StepOne Plus (Applied Biosystems, The Netherlands) or a Light Cycler 480 (Roche, Switzerland) instrument. Thermal cycling conditions included an initial denaturation step at 95°C for 3 min, followed by 45 cycles of 95°C for 15 s, 60°C for 30 s and 72°C for 30 s. In addition, a melting curve analysis of amplified products was performed between 55°C and 95°C. Cycle threshold (Ct) values below 40 and melting temperatures between 78.5 and 79.2°C were considered as CNM positive.

In order to further validate signals in positive samples, cDNA determined positive by detection of the *16S* rRNA gene was examined using a real-time PCR protocol targeting the CNM *groEL* gene using previously described methods [22].

For phylogenetic analysis, a 1262 bp of the CNM *16S* rRNA gene was sequenced, employing a nested PCR method including specific primers (Table 1). Thermal conditions included incubation at 94°C for 5 min, followed by 45 cycles at 95°C for 30 s, 58°C (PCR1) or 55°C (PCR2) for 30 s and 72°C for 3 min, and a final 7 min elongation at 72°C. Amplifications were performed using the Expand High FidelityPLUS PCR System (Roche, Switzerland) according to the manufacturer’s protocol, by using 2 μl of cDNA sample for the first PCR, and by diluting the resulting PCR product 100 times. One μl of this dilution was used as template in the second PCR. Amplified products were purified and sequenced by the Sanger method (Macrogen Europe, The Netherlands).

## Results and Discussion

A total of 5,365 birds were captured during the ringing season of 2009. Some birds were caught two or more times and each capture was counted separately. The captured birds represented 65 different species, of which 35 species were infested with one or more ticks. The most commonly infested bird species were tree pipit (*Anthus trivialis*, 87.7%), thrush nightingale (*Luscinia luscinia*, 78.4%) and common blackbird (*Turdus merula*, 64.8%) (Table 2). Of the 1,150 ticks that were screened for CNM, two were adult females, 582 were nymphs, 548 were larvae and 18 were undetermined (Table 3). Using morphological identification, 1,110 ticks were determined as *Ixodes* spp., 12 as *Haemaphysalis* spp. and 4 as *Hyalomma* spp. Genus identification was not possible in 24 ticks (Table 3). Since CNM was recorded only from nymphs, all nymphs and the two adult female ticks were further analysed by molecular methods. A total of 569 ticks (2 adults and 567 nymphs) were identified using *COI*. Of the analysed nymphs, 526 were identified as *I*. *ricinus*, five as *I*. *arboricola*, two as *Hyalomma marginatum* and four as *Ixodes* spp. Another 30 nymphs either did not amplify or did not give readable sequences despite several sequencing attempts. Thirty-nine ticks that could not be identified by *COI* were further screened using the *16S* rRNA PCR. This method identified 19 *I*. *ricinus* and 8 *I*. *frontalis* ticks. Six nymphs were identified as *Ixodes* spp. Four nymphs and one adult female could not be identified either by *COI* or *16S* rRNA PCR. The different results in the morphological and the molecular tick identification methods might be due to several reasons. As mentioned before, morphological identification of ticks from photographs is associated with uncertainties. Lv *et al*. developed a DNA barcoding system for Ixodida in 2013 [24] and they conclude that there still are some serious deficiencies in the information of *16S* and *COI* of some species of ticks. Furthermore, the performance of DNA barcoding can be influenced if species previously submitted to GenBank are incorrectly identified [25].

**Table 2:** Prevalence of tick infestation and *Candidatus* Neoehrlichia mikurensis in migratory birds captured at Ottenby, Sweden in 2009.

Bird species	No of birds examined for ticks*	Number of birds with tick	% of birds infested with ticks	Number of ticks screened for CNM	Number of CNM-positive ticks	% CNM-positive ticks
***Accipiter nisus***	5	1	20.0	1	0	0
***Acrocephalus arundinaceus***	1	0	0	0	0	0
***Acrocephalus palustris***	22	4	18.2	4	0	0
***Acrocephalus schoenobaenus***	23	0	0	0	0	0
***Acrocephalus scirpaceus***	11	1	9.1	1	0	0
***Anthus pratensis***	5	0	0	0	0	0
***Anthus trivialis***	106	93	87.7	85	1	1.2
***Carduelis cannabina***	15	1	6.7	1	0	0
***Carduelis carduelis***	15	0	0	0	0	0
***Carduelis chloris***	16	0	0	0	0	0
***Carduelis flammea***	41	7	17.1	7	3	42.9
***Carduelis spinus***	9	0	0	0	0	0
***Carpodacus erythrinus***	2	0	0	0	0	0
***Certhia familiaris***	28	4	14.3	4	0	0
***Coccothraustes coccothraustes***	1	0	0	0	0	0
***Corvus monedula***	1	0	0	0	0	0
***Delichon urbica***	43	0	0	0	0	0
***Dendrocopus major***	1	0	0	0	0	0
***Emberiza citrinella***	23	0	0	0	0	0
***Emberiza schoeniclus***	29	3	10.3	1	0	0
***Erithacus rubecula***	1796	619	34.4	513	6	1.2
***Ficedula albicollis***	2	0	0	0	0	0
***Ficedula hypoleuca***	31	0	0	0	0	0
***Ficedula parva***	8	0	0	0	0	0
***Fringilla coelebs***	39	8	20.5	8	0	0
***Fringilla montifringilla***	8	1	12.5	1	0	0
***Hippolais icterina***	50	2	4.0	2	0	0
***Hirundo rustica***	25	0	0	0	0	0
***Jynx torquilla***	1	0	0	0	0	0
***Lanius collurio***	43	3	7.0	3	0	0
***Locustella naevia***	1	0	0	0	0	0
***Luscinia luscinia***	37	29	78.4	29	2	6.9
***Luscinia svecica***	28	2	7.1	1	0	0
***Motacilla alba***	25	3	12.0	3	0	0
***Motacilla flava***	2	0	0	0	0	0
***Muscicapa striata***	55	0	0	0	0	0
***Oenanthe oenanthe***	6	1	16.7	1	0	0
***Parus caeruleus***	61	7	11.5	7	0	0
***Parus major***	49	8	16.3	2	0	0
***Passer domesticus***	16	1	6.3	1	0	0
***Passer montanus***	37	0	0	0	0	0
***Phoenicurus ochruros***	10	2	20.0	3	0	0
***Phoenicurus phoenicurus***	157	64	40.8	61	1	1.6
***Phylloscopus borealis***	1	0	0	0	0	0
***Phylloscopus collybita***	79	3	3.8	2	0	0
***Phylloscopus sibilatrix***	19	0	0	0	0	0
***Phylloscopus trochilus***	829	57	6.9	54	0	0
***Picus viridis***	1	0	0	0	0	0
***Prunella modularis***	48	20	41.7	17	1	5.9
***Pyrrhula pyrrhula***	2	0	0	0	0	0
***Regulus regulus***	319	9	2.8	8	0	0
***Saxicola rubetra***	1	0	0	0	0	0
***Serinus serinus***	2	0	0	0	0	0
***Sturnus vulgaris***	14	3	21.4	3	0	0
***Sylvia atricapilla***	58	4	6.9	4	0	0
***Sylvia borin***	42	0	0	0	0	0
***Sylvia communis***	145	49	33.8	44	1	2.3
***Sylvia curruca***	271	11	4.1	10	0	0
***Sylvia nisoria***	1	0	0	0	0	0
***Troglodytes troglodytes***	301	120	39.9	95	2	2.1
***Turdus iliacus***	42	23	54.8	21	1	4.8
***Turdus merula***	230	149	64.8	133	6	4.5
***Turdus philomelos***	73	22	30.1	20	0	0
***Turdus pilaris***	1	0	0	0	0	0
***Turdus viscivorus***	1	0	0	0	0	0
**Unknown (probably *Lanius collurio*)**	1	1	100.0	0	0	0
**Total**	**5365**	**1335**	**24.9**	**1150**	**24**	**2.1**

**Table 3:** CNM prevalence in different tick genera and tick stages. Genus identification is based on morphological identification. The numbers represent total number of ticks/ticks screened for CNM/CNM-positive ticks.

	*Adult female*	*Nymphs*	*Larvae*	*Un-determined*	*Total all stages*	*Total CNM-positive*	*CNM prevalence*
*Haemaphysalis* spp.	0	1/1/0	11/11/0	0	12/12/0	0	0%
*Hyalomma* spp.	1/1/0	2/2/0	2/1/0	0	5/4/0	0	0%
*Ixodes* spp.	1/1/0	613/579/24	589/528/0	3/2/0	1206/1110/24	24 nymphs	2.2%
*N/A*	0	3/0/0	11/8/0	98/16/0	112/24/0	0	0%
*Total number of ticks*	2/2/0	619/582/24	613/548/0	101/18/0	1335/1150/24	24 nymphs	2.1%
*CNM prevalence*	0%	4.1%	0%	0%			

**Fig 1:**
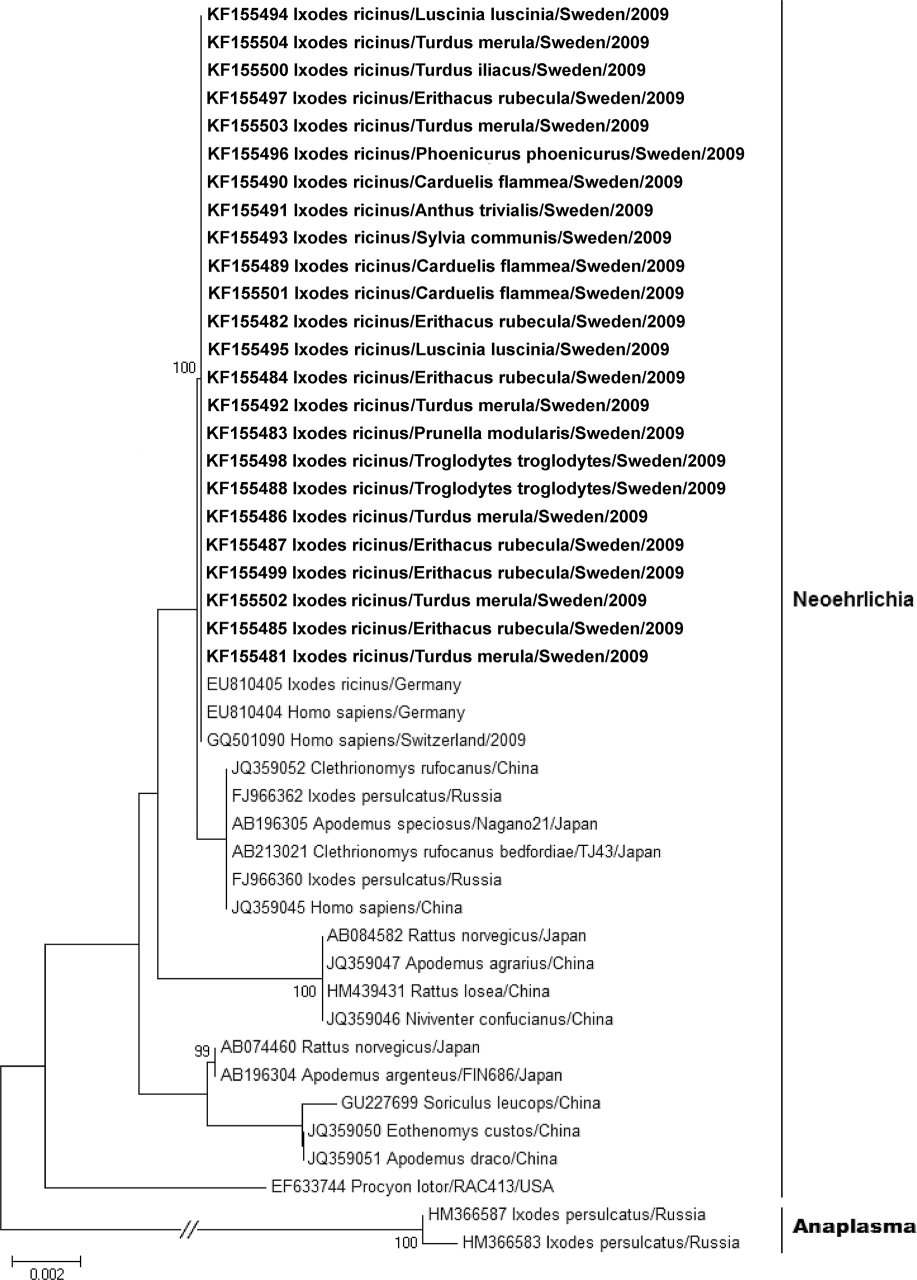
Phylogenetic relatedness of *Candidatus* Neoehrlichia mikurensis sequences. The tree is based on the partial nucleotide sequence of the *16S* rRNA gene of CNM and *Anaplasma phagocytophilum*. Bacterial sequences generated in this study (boldface) and previously reported sequences are denoted with GenBank accession number followed by information regarding sequence source. The tree was inferred using the neighbour joining method implemented in the SeaView software version 4 [27], utilizing 1000 bootstrap replications to determine support for inferred nodes. The tree was visualized using the MEGA software version 5.2 [28], and statistical supports of > 95% for inferred nodes are displayed. In the mid-point rooted tree, the branch between the CNM and the *Anaplasma* clade has been truncated in order to limit the tree size. The scale bar represents the number of substitutions per site. The CNM sequences were all 100% identical to each other as well as to three other European strains, including those detected in humans, and therefore appear in a single clade.

Forty-nine ticks (*i*.*e*. 4.3%) were positive for CNM when screened with the *16S-*based PCR method, and 31 of these remained positive after the *groEL*-PCR screening. Sequencing revealed that the established *16S* rRNA-PCR lacked somewhat in selectivity since it also detected bacteria other than CNM, such as *Rickettsia* spp., *Candidatus* Midichloria spp. and others. However, CNM sequences were obtained from 24 of the 31 samples that were positive in screenings with both the *16S* and the *groEL* method. Alignment and phylogenetic analysis [26] showed that the CNM sequences were identical to each other as well as to three other European strains, including those detected in humans, and appeared in a single clade (Fig 1). Thus, based on bacterial gene sequence identification, 2.1% (24/1150) of analysed ticks were concluded to be CNM positive. This might be an underestimation of the true prevalence, since the CNM RNA level in the samples could be too low to be detected.

According to molecular species identification, *I*. *ricinus* was the dominant tick species collected from the birds in this study. The CNM-infected ticks were all nymphs and 22 of them were identified as *I*. *ricinus* (Table 4). The remaining two CNM-infected ticks were morphologically identified as *I*. *ricinus*, but were not subjected to molecular testing. The prevalence of CNM in molecularly identified *I*. *ricinus* nymphs was 4.2% (22/526) (Table 3). None of the 548 larvae was positive for CNM.

**Table 4:** *Candidatus* Neoehrlichia mikurensis-positive tick nymphs from birds in Sweden 2009.

	Bird no	Bird species	Migration	Collection month	Tick species ^§^	Feeding status	Tick location
**Spring**	1	*Turdus merula*	SD*	March	*I*. *ricinus*	HF	Bill
	2	*Turdus merula*	SD	April	*I*. *ricinus*	LF	Bill
	3	*Anthus trivialis*	SD	April	*I*. *ricinus*	HF	Bill
	4	*Erithacus rubecula*	SD	April	*I*. *ricinus*	FF	Bill
	5	*Prunella modularis*	SD	April	*I*. *ricinus*	U	Bill
	6	*Erithacus rubecula*	SD	April	*I*. *ricinus*	FF	Bill
	7	*Erithacus rubecula*	SD	April	*I*. *ricinus*	FF	Bill
	8	*Erithacus rubecula*	SD	April	*I*. *ricinus*	LF	Bill
	9†	*Carduelis flammea*	SD	April	*I*. *ricinus*	LF	Eye
	9†	*Carduelis flammea*	SD	April	*I*. *ricinus*	HF	Eye
	10	*Troglodytes troglodytes*	SD	April	*I*. *ricinus*	HF	Bill
	11	*Turdus merula*	SD	May	*I*. *ricinus*	HF	Bill
**Autumn**	12	*Sylvia communis*	LD	August	*I*. *ricinus*	LF	Bill
	13†	*Luscinia luscinia*	LD	August	*I*. *ricinus*	FF	Ear
	13†	*Luscinia luscinia*	LD	August	*I*. *ricinus*	HF	Bill
	14	*Phoenicurus phoenicurus*	LD	August	*I*. *ricinus*	HF	Bill
	15	*Erithacus rubecula*	SD	September	*I*. *ricinus*	U	Bill
	16	*Troglodytes troglodytes*	SD	September	*I*. *ricinus*	HF	Bill
	17	*Turdus merula*	SD	October	*I*. *ricinus*	FF	Eye
	18	*Turdus merula*	SD	October	*I*. *ricinus*	HF	Bill
	19	*Turdus merula*	SD	October	*I*. *ricinus*	HF	Eye
	20	*Turdus iliacus*	SD	October	N/A	HF	Bill
	21	*Erithacus rubecula*	SD	October	*I*. *ricinus*	FF	Bill
	22	*Carduelis flammea*	SD	November	N/A	U (-LF)	Bill

§ Tick species was molecularly identified by *COI* and *16S*.

* SD = short-distance migrant, HF = half fed, LF = little fed, FF = fully fed, U = unfed (no blood), LD = long-distance migrant (trans-Mediterranean or trans-Saharan)

† *=* bird that carried two *Candidatus* Neoehrlichia mikurensis-positive ticks.

This study contains the largest amount of ticks collected from birds that has been screened for CNM to date. The highest CNM-prevalences were recorded in nymphs collected from common redpoll (*Carduelis flammea*, 3/7 = 42.9%), thrush nightingale (*Luscinia luscinia*, 2/29 = 6.9%) and dunnock (*Prunella modularis*, 1/17 = 5.9%) (Table 2). The finding that CNM was recorded only from nymphs, and not from larvae, corresponds well with earlier studies and may suggest that CNM is not transovarially transmitted [8, 14, 15]. The majority of the larvae was blood-fed; they would thus presumably have acquired their infection from the bird they were feeding on. All CNM-positive ticks were identified as *I*. *ricinus*, a species which has a very wide host-feeding range. Strictly ornithophagous tick species were also found, but in low numbers and none was found positive for CNM. In this study, we found two individual birds which were infested by two CNM-positive nymphs respectively (Table 4). The first bird, a common redpoll, was infested with a total of five nymphs. Four of them were molecularly identified as *I*. *ricinus* and no sequence was obtained from the fifth, but it was morphologically identified as *I*. *ricinus*. Both CNM-positive nymphs were located at the bill of the bird, one was little fed and one was half fed (Table 4). The second bird was a thrush nightingale. It was infested with a total of four molecularly identified *I*. *ricinus* nymphs and three larvae morphologically identified as *Ixodes* spp. Two of the nymphs were CNM-positive; one of them fully engorged and located at the ear, the other one half fed and located at the bill of the bird. In the redpoll, the CNM-positive nymphs were attached adjacent to each other, which may indicate that CNM might be transferred among ticks co-feeding close together on the same host. The CNM-positive ticks on these two birds were all nymphs and the possibility that they independently acquired the organism as larvae cannot be ruled out. However, there are only few records of a CNM-positive larva so far [29]. Alternatively, these ticks had been infected while feeding on a systemically infected bird. However, the presence of several other CNM-negative, fully or partially engorged nymphs on the redpoll makes this assumption less likely. Unfortunately, no blood or tissue samples were collected from the tick-infested birds.

Information on possible vectors of CNM is of importance for understanding the geographical distribution of this pathogen. It is still unclear whether birds can act as reservoirs for CNM. A recent study indicated that birds bacteraemic with *Rickettsia helvetica* or *Anaplasma phagocytophilum* seem capable to infect ticks feeding on them [30].

The CNM-positive ticks were collected from 10 different bird species, all of which were predominantly ground foraging species, and thus more intensively exposed to ticks than birds within a more arboreal ecological guild. CNM was found in *I*. *ricinus* both during the avian hosts’ spring and autumn migrations. CNM-positive ticks were predominantly detected on short-distance migrant birds that spend the winter in Europe. Only few CNM-positive ticks were found on trans-Mediterranean or trans-Saharan migrants (Table 4). However, the CNM-infected ticks attached to these long-distance migrants were only collected during autumn, implying that these birds had become tick-infested in Northern Europe.

In conclusion, even though our material contained a large amount of blood-fed larvae, CNM was found only in nymphs and the prevalence in molecularly determined *I*. *ricinus* nymphs was 4.2%. This corresponds well with recent findings from Switzerland, where 3.3% of ticks collected from birds were CNM-positive, all of them nymphs [14]. This study shows that birds most likely can disperse CNM-infected ticks over large geographical areas. Different bird species may differ in their potential to disperse CNM. Their CNM dispersal potential should be influenced by their genetically determined migration patterns and other intrinsic behaviour patterns such as if they tend to feed on the ground or not.

## References

[1] Kawahara M, Rikihisa Y, Isogai E, Takahashi M, Misumi H, Suto C, et al Ultrastructure and phylogenetic analysis of '*Candidatus* Neoehrlichia mikurensis' in the family *Anaplasmataceae*, isolated from wild rats and found in *Ixodes ovatus* ticks. Int J Syst Evol Microbiol. 2004;54(Pt 5):1837–43. 15388752 10.1099/ijs.0.63260-0

[2] Schouls LM, Van De Pol I, Rijpkema SG, Schot CS. Detection and identification of *Ehrlichia*, *Borrelia burgdorferi* sensu lato, and *Bartonella* species in Dutch *Ixodes ricinus* ticks. J Clin Microbiol. 1999;37(7):2215–22. 10364588 10.1128/jcm.37.7.2215-2222.1999 PMC85121

[3] Welinder-Olsson C, Kjellin E, Vaht K, Jacobsson S, Wenneras C. First case of human "*Candidatus* Neoehrlichia mikurensis" infection in a febrile patient with chronic lymphocytic leukemia. J Clin Microbiol. 2010;48(5):1956–9. 10.1128/JCM.02423-09 20220155 PMC2863919

[4] Grankvist A, Andersson PO, Mattsson M, Sender M, Vaht K, Hoper L, et al Infections with the tick-borne bacterium "*Candidatus* Neoehrlichia mikurensis" mimic noninfectious conditions in patients with B cell malignancies or autoimmune diseases. Clin Infect Dis. 2014;58(12):1716–22. 10.1093/cid/ciu189 24647019

[5] Welc-Falęciak R, Siński E, Kowalec M, Zajkowska J, Pancewicz SA. Asymptomatic "*Candidatus* Neoehrlichia mikurensis" Infections in Immunocompetent Humans. J Clin Microbiol. 2014;52(8):3072–4. 10.1128/JCM.00741-14 24899023 PMC4136151

[6] Andréasson K, Jönsson G, Lindell P, Gülfe A, Ingvarsson R, Lindqvist E, et al Recurrent fever caused by *Candidatus* Neoehrlichia mikurensis in a rheumatoid arthritis patient treated with rituximab. Rheumatology (Oxford). 2014. 10.1093/rheumatology/keu441 25416710

[7] Li H, Jiang JF, Liu W, Zheng YC, Huo QB, Tang K, et al Human Infection with *Candidatus* Neoehrlichia mikurensis, China. Emerg Infect Dis. 2012;18(10):1636–9. 10.3201/eid1810.120594 23017728 PMC3471638

[8] Jahfari S, Fonville M, Hengeveld P, Reusken C, Scholte EJ, Takken W, et al Prevalence of *Neoehrlichia mikurensis* in ticks and rodents from North-west Europe. Parasit Vectors. 2012;5(1):74. 22515314 10.1186/1756-3305-5-74 PMC3395572

[9] Maurer FP, Keller PM, Beuret C, Joha C, Achermann Y, Gubler J, et al Close geographic association of human neoehrlichiosis and tick populations carrying "*Candidatus* Neoehrlichia mikurensis" in eastern Switzerland. J Clin Microbiol. 2013;51(1):169–76. 10.1128/JCM.01955-12 23115262 PMC3536216

[10] Richter D, Kohn C, Matuschka FR. Absence of *Borrelia* spp., *Candidatus* Neoehrlichia mikurensis, and *Anaplasma phagocytophilum* in questing adult *Dermacentor reticulatus ticks* . Parasitology research. 2013;112(1):107–11. 10.1007/s00436-012-3110-8 22955502

[11] Vayssier-Taussat M, Le Rhun D, Buffet JP, Maaoui N, Galan M, Guivier E, et al *Candidatus* Neoehrlichia mikurensis in bank voles, France. Emerg Infect Dis. 2012;18(12):2063–5. 10.3201/eid1812.120846 23171720 PMC3557860

[12] Andersson M, Råberg L. Wild Rodents and Novel Human Pathogen *Candidatus* Neoehrlichia mikurensis, Southern Sweden. Emerg Infect Dis. 2011;17(9):1716–8. 10.3201/eid1709.101058 21888802 PMC3322053

[13] Hasle G. Transport of ixodid ticks and tick-borne pathogens by migratory birds. Front Cell Infect Microbiol. 2013;3:48 10.3389/fcimb.2013.00048 24058903 PMC3767891

[14] Lommano E, Dvorak C, Vallotton L, Jenni L, Gern L. Tick-borne pathogens in ticks collected from breeding and migratory birds in Switzerland. Ticks Tick Borne Dis. 2014;5(6):871–82. 10.1016/j.ttbdis.2014.07.001 25113989

[15] Spitalská E, Literák I, Sparagano OA, Golovchenko M, Kocianová E. Ticks (Ixodidae) from passerine birds in the Carpathian region. Wien Klin Wochenschr. 2006;118(23–24):759–64. 17186172 10.1007/s00508-006-0729-4

[16] Movila A, Alekseev AN, Dubinina HV, Toderas I. Detection of tick-borne pathogens in ticks from migratory birds in the Baltic region of Russia. Med Vet Entomol. 2012. 10.1111/j.1365-2915.2012.01037.x 22924442

[17] Arthur DR. British ticks. London: Butterworths; 1963.

[18] Filippova NA. Ixodid ticks of the subfamily Ixodinae 4th Edition ed. Leningrad: Nauka; 1977. 396 p.

[19] Hillyard PD. Ticks of North-West Europe: Keys and notes for identification of the species Synopses of the British Fauna. New Series no 52 Edited by Banks R.S.K. and Crothers J. H.. Shrewsbury: The Linnean Society of London and The Estuarine and Coastal Sciences Association; 1996.

[20] Heylen D, De Coninck E, Jansen F, Madder M. Differential diagnosis of three common Ixodes spp. ticks infesting songbirds of Western Europe: *Ixodes arboricola*, *I*. *frontalis* and *I*. *ricinus* . Ticks Tick Borne Dis. 2014;5(6):693–700. 10.1016/j.ttbdis.2014.05.006 25113983

[21] Lv J, Wu S, Zhang Y, Chen Y, Feng C, Yuan X, et al Assessment of four DNA fragments (COI, 16S rDNA, ITS2, 12S rDNA) for species identification of the Ixodida (Acari: Ixodida). Parasit Vectors. 2014;7:93 10.1186/1756-3305-7-93 24589289 PMC3945964

[22] Andersson M, Bartkova S, Lindestad O, Raberg L. Co-infection with '*Candidatus* Neoehrlichia mikurensis' and *Borrelia afzelii* in *Ixodes ricinus* ticks in southern Sweden. Vector borne and zoonotic diseases. 2013;13(7):438–42. 10.1089/vbz.2012.1118 23590321

[23] Wilhelmsson P, Lindblom P, Fryland L, Ernerudh J, Forsberg P, Lindgren PE. Prevalence, Diversity, and Load of *Borrelia* species in Ticks That Have Fed on Humans in Regions of Sweden and Åland Islands, Finland with Different Lyme Borreliosis Incidences. PLoS One. 2013;8(11):e81433 10.1371/journal.pone.0081433 24278437 PMC3836827

[24] Lv J, Wu S, Zhang Y, Zhang T, Feng C, Jia G, et al Development of a DNA barcoding system for the Ixodida (Acari: Ixodida). Mitochondrial DNA. 2014;25(2):142–9. 10.3109/19401736.2013.792052 23631370

[25] Zhang RL, Zhang B. Prospects of using DNA barcoding for species identification and evaluation of the accuracy of sequence databases for ticks (Acari: Ixodida). Ticks Tick Borne Dis. 2014;5(3):352–8. 10.1016/j.ttbdis.2014.01.001 24656809

[26] Saitou N, Nei M. The neighbor-joining method: a new method for reconstructing phylogenetic trees. Mol Biol Evol. 1987;4(4):406–25. 3447015 10.1093/oxfordjournals.molbev.a040454

[27] Gouy M, Guindon S, Gascuel O. SeaView version 4: A multiplatform graphical user interface for sequence alignment and phylogenetic tree building. Mol Biol Evol. 2010;27(2):221–4. 10.1093/molbev/msp259 19854763

[28] Tamura K, Peterson D, Peterson N, Stecher G, Nei M, Kumar S. MEGA5: molecular evolutionary genetics analysis using maximum likelihood, evolutionary distance, and maximum parsimony methods. Mol Biol Evol. 2011;28(10):2731–9. 10.1093/molbev/msr121 21546353 PMC3203626

[29] Derdáková M, Václav R, Pangrácova-Blaňárová L, Selyemová D, Koči J, Walder G, et al Candidatus Neoehrlichia mikurensis and its co-circulation with *Anaplasma phagocytophilum* in *Ixodes ricinus* ticks across ecologically different habitats of Central Europe. Parasit Vectors. 2014;7:160 10.1186/1756-3305-7-160 24693971 PMC3984398

[30] Hornok S, Kováts D., Csörgo T., Meli M.L., Gönczi E., Hadnagy Z., Takács N., Farkas R., Hofmann-Lehmann R. Birds as potential reservoirs of tick-borne pathogens: first evidence of bacteraemia with *Rickettsia helvetica* . Parasit Vectors. 2014;7(128). 10.1186/1756-3305-7-128 PMC3976504 24679245

